# Influence of learning activities and background characteristics on pharmacology exam success in second-year medical students at a French university: the Pharmaquest study

**DOI:** 10.1186/s12909-026-09454-7

**Published:** 2026-05-18

**Authors:** Jean-Marie Dumont, Basile Chretien, Pierre-Marie Morice, Eric Saillot, Xavier Humbert, Damien Legallois, Branko Aleksic, Hideki Kasuya, Sophie Fedrizzi, Joachim Alexandre, Véronique Lelong-Boulouard, Charles Dolladille

**Affiliations:** 1 Université Caen Normandie, UNICAEN, CEMU, Service d’Appui à la Pédagogie, Caen, F-14000 France; 2 Department of International Medical Education, Nagoya University Graduate School of Medicine, Nagoya, Aichi Japan; 3 Normandie University, UNICAEN, INSERM, U1237, Neuropresage Team, Cyceron, Caen, F-14000 France; 4 Department of Pharmacology, Normandie University, UNICAEN, INSERM U1086 ANTICIPE, Caen, F-14000 France; 5 Normandie University, UNICAEN, CIRNEF, UR7454, Caen, F-14000 France; 6 Department of General Practice, Normandie University, UNICAEN, Caen, France; 7 Department of Cardiology, Caen-Normandy University Hospital, Caen, France; 8 Department of Pharmacology, Caen-Normandy University Hospital, Caen, France; 9 Normandie University, UNICAEN, INSERM U1075 COMETE, GIP CYCERON, Caen, F-14000 France; 10 Pharmacology Department, Caen University Hospital, Caen, 14033 France

**Keywords:** Pharmacology education, Academic performance, Pedagogical methods, Academic background, Medical students

## Abstract

**Background:**

Pharmacology education faces challenges due to evolving student learning behaviors. This study examined the feasibility of identifying which student-level characteristics, academic background and learning activities were associated with performance at the pharmacology exam using routinely-collected data.

**Methods:**

In a prospective cohort study of second-year medical students at Caen Normandie University (2024–2025), we analyzed in-person lecture, online resource, and peer tutoring mock exam attendance, as well as use of student-written lecture support, and pharmacology exam outcomes. The primary outcome was final pharmacology exam score (over 20points), according to pedagogical methods, estimated through multivariate linear regression.

**Results:**

Participants (164 out-of 250 eligible students(66%)) were well representative of 2^nd^year medical students, with two thirds of women, high prior academic achievement, and a median age of 19. In the univariate analysis, higher in-person lecture attendance was significantly associated with a higher score at the pharmacology exam (+ 0.41points for each additional 10h of attendance, 95%Confidence Interval (CI): + 0.05 to + 0.77). Similarly, the number of online resources downloaded showed a positive association in univariate analysis (+ 0.53points for each additional 10downloads, 95%CI: + 0.09 to + 0.97). In-person lecture attendance remained significant in the multivariate model (+ 0.46points, 95%CI:0.03–0.88). Peer tutoring exam attendance and student-written lecture support use were not associated with a significant change in exam score.

In-person lecture attendance exhibited a U-shaped relationship with exam performance: initial declines (up to 10h) preceded gradual improvement, plateauing at 25–30 h (spline regression, *p* < 0.05). Prior academic achievement (baccalaureate distinction, rapid first-year completion) and curriculum (first-year major being health-science) were significantly associated with higher exam score.

**Conclusions:**

This pilot study demonstrates the feasibility and value of quantitatively evaluating pharmacology education among French medical students. Academic background and pedagogical methods seem to influence pharmacology exam success. Broader replication, and long-term follow-up are needed to confirm these insights and identify determinants of student success more robustly.

**Supplementary Information:**

The online version contains supplementary material available at 10.1186/s12909-026-09454-7.

## Background

Pharmacology is a core discipline in medical education, essential not only for academic success but also for clinical competence and patient safety [1]. Yet many medical students struggle to master it. The growing volume and complexity of pharmacological knowledge, combined with limited time, increasing curricular pressure, and a growing number of attending students have challenged traditional teaching methods for today’s learners.

New generations of students are adopting diverse and tech-enabled learning behaviors—ranging from passive lecture attendance to active use of digital tools [2]. A noticeable decline in Lecture-based learning (LBL, traditional lecture) attendance has been observed over time among medical students, likely reflecting a mismatch between conventional teaching methods and evolving student preferences and needs [3, 4]. This shift has prompted educators to explore more engaging approaches, including flipped classrooms, problem-based learning, and serious games [5, 6]. These active learning strategies have shown promise in improving understanding and retention in pharmacology education [7]. However, the alignment between these pedagogical innovations and students’ actual learning behaviors remains poorly understood. Furthermore, while previous studies have examined the role of demographic factors in academic success [8], the specific predictors of pharmacology performance—particularly pedagogical methods and educational background—remain underexplored. Existing studies in India and Sweden suffer from limited methodological rigor: reliance on dichotomous outcomes (e.g., pass/fail), simplistic models, and/or a lack of adjustment for key confounders [9, 10]. Interpreting and generalizing findings across educational systems must be approached with caution: differences in the structure and delivery of pharmacology education, as well as cultural and institutional contexts, can significantly shape learning outcomes. This gap is especially critical in the context of France’s recent reform of medical school admissions. Since 2020, France has replaced its traditional single competitive entrance year for health studies with two new admission pathways: the *Parcours Accès Santé Spécifique* (PASS) and the *Licence avec Accès Santé* (L.AS). PASS is a health-focused track that integrates a major in health sciences with a minor in another discipline, while L.AS corresponds to a general bachelor’s degree (e.g., biology, law, psychology) complemented by a health module enabling students to apply to MMOPK programs (Medicine, Midwifery, Odontology, Pharmacy, and Physiotherapy) [11]. These reforms were implemented to reduce the high-stakes nature of the previous competitive examination, limit the prevalence of repeat years, encourage broader academic backgrounds, and promote equity in access. Consequently, admitted students now display greater heterogeneity not only in prior scientific knowledge (particularly in chemistry, biology, and mathematics) but also in study methods, time management strategies, and familiarity with self-directed learning. Although this diversification may enrich group dynamics and learning approaches, its impact on specific disciplines—such as pharmacology, which requires both scientific grounding and clinical reasoning—remains largely unexplored. Although the curricular structure differs across countries, the underlying question, which student behaviours and academic background factors actually translate into pharmacology performance, is of universal interest to educators.

To address these gaps, we designed *Pharmaquest*—a prospective pilot study on second-year medical students at Caen Normandy University in France. We investigated how learning activities (in-person lecture attendance, online resource use, peer-tutoring mock exam attendance, and use of student-written lecture support) and student background characteristics influenced pharmacology exam performance, and explored the contribution of different in-person pedagogical formats.

## Methods

### Study design and setting

Pharmaquest was a prospective, monocentric, observational cohort study on second-year medical students conducted at Caen Normandy University in France during the 2024–2025 academic year. The study protocol was prospectively registered on Zenodo [12], and approved by the institutional ethics committee (approval number 2024053017261400000110000546). All data were pseudonymized and linked using randomly generated identifiers to preserve student confidentiality.

### Participants

All second-year (i.e. second license year) medical students were eligible. Participation was voluntary, and informed consent was obtained from all participants. Participating to the Pharmaquest study did not result in any benefit or harm regarding the pharmacology exam, as teachers were unaware of student’s participation before releasing the exam score. Eligibility criteria included adult (18 years old or older) students. Non-inclusion criteria included prior exposure to pharmacology courses and lack of internet access, and exclusion criteria was the non-presentation at the final pharmacology exam.

### Data collection

Data were collected from multiple sources to comprehensively assess students’ academic background, pedagogical methods used, and pharmacology performance. Learning activities consisted in in-person lecture, and peer tutoring attendance, use of online resources, and use of student-written lecture support (hereafter, roneo [13]).

#### Demographic and academic background

Age, gender, distinction awarded at the French *baccalauréat* (baccalaureate)—a national secondary school leaving examination and diploma awarded at the end of high school, which grants access to higher education—first license year (the first year of the bachelor-equivalent French university curriculum granting access to health-science studies (PASS / L.AS pathway)) major subject (one of biology, chemistry, economy, health sciences, informatics, law, mathematics, psychology, physics, sport sciences, or other), and number of years taken to complete the first license year were collected at baseline directly from participants. The time to complete the first license year variable was coded as 0, 1, 2, or 3 years, where “0 years” identifies students who entered second-year medical studies via the dedicated transition programme (“passerelle”) without spending a year in PASS or L.AS, typically students holding a prior science Master’s or doctoral degree. The *baccalauréat* is graded on a scale from 0 to 20, and students may receive an overall distinction based on their final score: no distinction (score < 12), fair (12–13.9), good (14–15.9), and very good (≥ 16). This distinction was included in the analysis as a proxy for students’ academic achievement prior to entering university and provides an internationally understandable measure of prior academic performance.

##### In-person lectures

Lectures were provided by 5 senior pharmacology educators, either physicians or pharmacists, for a total of 34h of classes. Each lecture was categorized into a set of possible pedagogical format types (LBL, problem-based learning, serious game, evidence-based medicine, flipped classrooms, team-based learning, case-based learning, see Supplementary Table 1 for details) by a single author (CD) interviewing his colleagues. A lecture could be classified into one or many types. We recorded the date, time, duration, name and type of each lecture, as well as the educator. Lectures took place between September and November, 2024. Attendance to in-person lectures was optional, and monitored using a voluntary QR code system. At the beginning of each lecture, students could scan a QR code displayed in the classroom using their personal devices, which logged their attendance in an online form based on limesurvey [14].

##### Online resources

Access data from the university’s learning management system (Moodle, version 4.1 [15]) were extracted directly from the platform’s event log using SQL queries based on predefined criteria. The data included the number and type of resources accessed (e.g., lecture support materials, Teacher’s forum interactions, external links). The in-person lecture supports were uploaded immediately after each lecture to the online platform and were not available to students beforehand. During the data processing, each online resource was accounted only once per student, irrespective of the number of consultations.

##### Peer tutoring mock exam

A peer tutoring service is organized by the student association. Third-year medical students (L3) prepare mock exams for second-year students (L2), mimicking the format and content of the actual pharmacology exams. These mock exams may be reviewed by faculty members from the relevant disciplines, which was the case for pharmacology (CD). In the 2024–2025 academic year, the pharmacology tutoring session consisted exclusively of multiple-choice questions (MCQs). The L2 student’s office, responsible for overseeing the MCQs correction process, retained records of individual student scores. These records were used to retrieve both attendance and performance data related to the tutoring session. The tutoring session took place on November, 2024, later than the last in-person lecture.

##### Roneo lecture support access

The student association organizes a service of student-written lecture support, to complement in-person lectures and official lecture supports. These supports are drafted by 2 students attending the in-person lecture. Other students may access these supports, provided they pay for a subscription.

##### Pharmacology performance – exam scores

A summative exam was the sole assessment for the pharmacology teaching unit titled *Bases Moléculaires, Cellulaires et Tissulaires des Thérapeutiques* (BMCT, or *Molecular, Cellular, and Tissue Bases of Drug Treatments*). The exam follows the national guidelines for dematerialized evaluations [16]. It consists of 40 isolated questions using a variety of formats including MCQs, Single Best Answers (SBAs), Very Short Answer Questions (VSAQs), and List-based responses. Each course within the unit is represented by at least one question. Questions were strictly derived from the official lecture materials provided to students, ensuring alignment between assessment and taught content.

The questions are drafted by the respective instructors responsible for each lecture and are then reviewed and approved by the pharmacology unit coordinator. The finalized exam paper is locked approximately one month prior to the exam session. Final results were normalised to a single score on a 20-point scale, with higher values indicating better performance; the maximum achievable score was therefore 20, in accordance with standard academic grading in France.

Following the exam, items with a success rate below 20% are systematically reviewed by the original authors to ensure alignment with course content and absence of ambiguity or error. Items identified as problematic may be cancelled. Final results consist in a single score, over 20points, with higher scores indicating a higher performance at the exam. The exam was passed on December, 2024.

### Outcomes

The primary outcome was the association between learning activities and the final pharmacology exam score.

Secondary outcomes included:

- The association between class attendance and the final pharmacology exam score, including assessment of potential non-linear relationships
- The association between the final pharmacology exam score and:
  ◦ Class format types
  ◦ Online resource types
  ◦ Academic background (baccalaureate distinction, first license year major, time to complete the first license year)
  ◦ Age and sex
- The correlation between the pharmacology peer tutoring mock exam score and the final pharmacology exam score.
- The feasibility of quantitatively evaluating a pharmacology educational program within the context of French academic medical training.

### Statistical analysis

To assess the primary outcome, a multivariable linear regression model was used, adjusting for potential confounders. The multivariable model was pre-specified before analysis: variables included the two exposures of interest (total in-person lecture attendance in hours and number of distinct online resources accessed) and a fixed set of conceptually-driven confounders (age, sex, baccalaureate distinction, first-year licence major, time to complete the first license year, roneo subscription, and peer-tutoring mock-exam participation). No data-driven variable selection (e.g., stepwise procedures) was performed. Model assumptions (linearity, homoscedasticity, normality of residuals) were assessed, and multicollinearity was evaluated using variance inflation factors (VIF).

To examine a potential non-linear relationship between class attendance and exam performance, we used spline regression (natural splines with varying degrees of freedom). An exploratory Random Forest model (500 trees) was also applied to identify complex interactions and assess variable importance. These were interpreted using SHAP (Shapley Additive Explanations) values, which quantify the contribution of each feature to the model’s predictions by considering all possible combinations of feature inputs (see Supplementary Methods).

For the association analyses between exam score and class format types or online resource types, we applied univariate linear regression models with confidence intervals to assess precision.

The correlation between the pharmacology peer tutoring mock exam score and the final pharmacology exam score was calculated using the Pearson correlation coefficient.

Categorical variables were summarized using frequency and percentage, and continuous variables using median and interquartile range (IQR). All statistical tests were two-sided with a significance level of α = 0.05. Analyses were performed in R (version 4.4.3) using the randomForest, shapviz, fastshap, and splines packages.

## Results

**Fig. 1:**
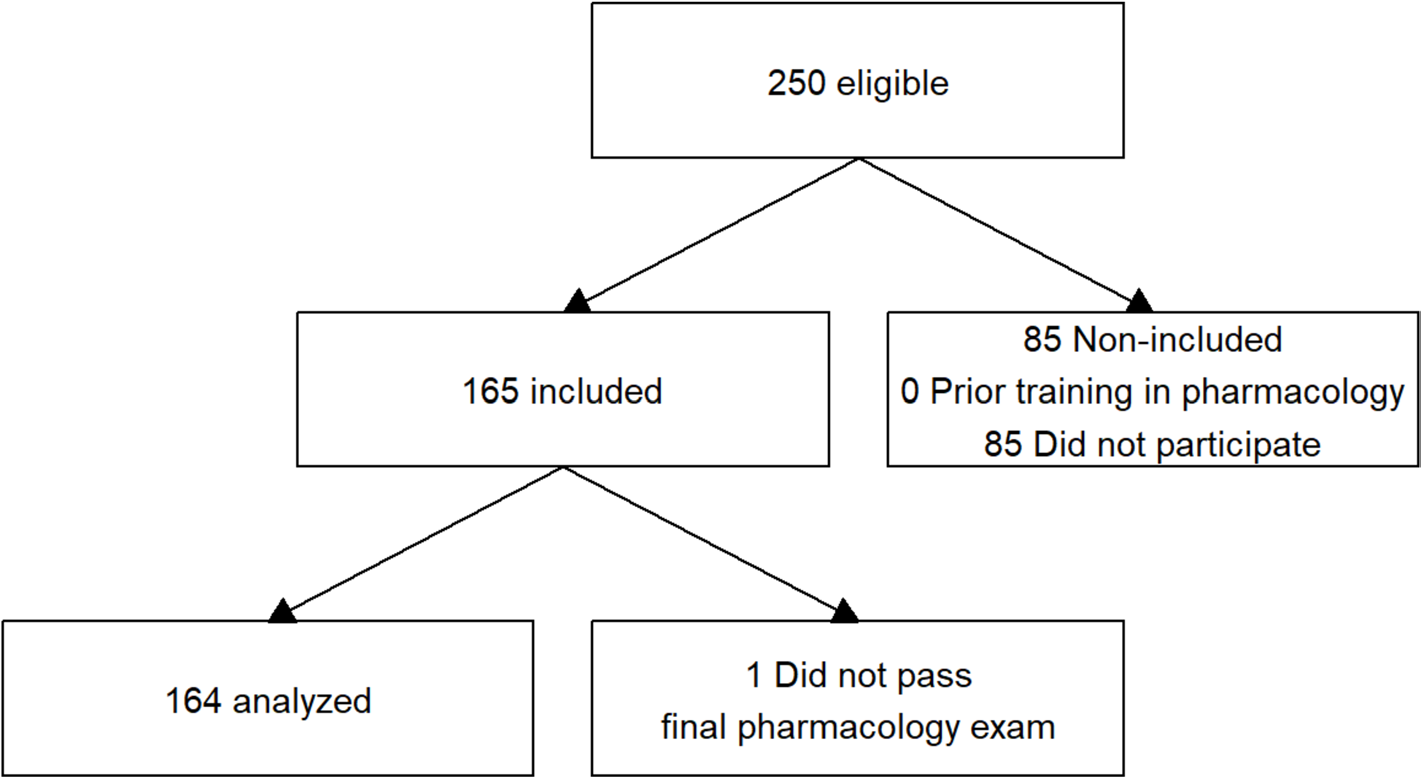
Study flowchart. Of the 250 eligible second-year medical students, 85 declined consent. Because data were pseudonymised, no individual-level information was retained for non-consenting students; the reason for non-participation was not recorded. One participant did not attend the final pharmacology exam and was therefore not included in the primary analysis

Of the 250 second-year medical students enrolled at Caen Normandie University, 165 consented to participate in the study (85 declined), and 164 were included in the final analysis, as one participant did not attend the final pharmacology examination (see Fig. 1). Compared with the aggregate characteristics of the full second-year cohort (*n* = 250), participants had a similar median age and a similar proportion of women, suggesting that the participating sample was broadly representative of the source population. Because of the pseudonymisation procedure, individual-level data on non-consenting students were not available, and a direct comparison of pharmacology exam scores between consenters and non-consenters could not be performed.

### Baseline characteristics

#### Students

Characteristics of the participating students are presented in Table 1. The median age of participants was 19 years (standard deviation: 2.4), and the majority were female (*n* = 105, 64.4%). Regarding academic background, only 6 students (3.7%) did not receive a distinction on the baccalaureate, while 124 (75.6%) obtained either a good or very good distinction. The most common first license year majors were Health Sciences (*n* = 76, 46.3%), Life Sciences (*n* = 19, 11.6%), and Psychology (*n* = 18, 11.0%). Most students completed the first year of medical studies within one or two years (*n* = 149, 90.9%); five students (3.0%) were included in the “0 years” group, as they entered directly into the second year via a bridging program.

**Table 1:** Baseline student’s characteristics of second year medical students participating to Pharmaquest

Variable	Stats / Values	Freqs (% of Valid)	Graph	Valid	Missing
Age	Mean (SD): 19.8 (2.4) min ≤ med ≤ max: 18 ≤ 19 ≤ 36 IQR (CV): 1 (0.1)	12 distinct values		164 (100.0%)	0 (0.0%)
Gender	Female Male	105(64.4%) 58(35.6%)		163 (99.4%)	1 (0.6%)
Distinction to the baccalaureate	No distinction Fairly good Good Very good	6(3.7%) 34(20.7%) 59(36.0%) 65(39.6%)		164 (100.0%)	0 (0.0%)
First license year major	Other Chemistry Economy Law Informatics Mathematics Physic Psychology Life sciences Health sciences Sport sciences	5(3.0%) 8(4.9%) 6(3.7%) 6(3.7%) 4(2.4%) 6(3.7%) 4(2.4%) 18(11.0%) 19(11.6%) 76(46.3%) 12(7.3%)		164 (100.0%)	0 (0.0%)
Number of license years spent before accessing 2nd year medical school	Mean (SD): 1.4 (0.7) min ≤ med ≤ max: 0 ≤ 1 ≤ 3 IQR (CV): 1 (0.5)	0:5(3.0%) 1:91(55.5%) 2:58(35.4%) 3:10(6.1%)		164 (100.0%)	0 (0.0%)

*CV* Coefficient of Variation, *Freqs* Frequencies, *IQR* Interquartile range, *med* median, *min* minimum, *max* maximum

#### Courses

Students attended a median of 8 lectures (range 0–31) and downloaded a median of 9 unique online resources (range 0–25). Nearly all students (95%) subscribed to the Roneo lecture support, and 54% participated in the peer-tutoring mock exam (Table 2). Student participation throughout the semester revealed a progressive decline in in-person lecture attendance, (Supplementary Fig. 1). Almost all lectures included LBL. Case-based learning (CBL; 4 sessions), problem-based learning (PBL; 3), serious games (3), evidence-based medicine (EBM; 2), team-based learning (TBL; 2), and flipped classrooms (1) were also used (Supplementary Table 1). A total of 22 lecture supports, 2 external resource links, and 3 subjects on the teacher’s forum were made available online. As shown in Supplementary Fig. 2, students primarily interacted with the lecture supports, which received 2,931 clicks—an average of 17.8 clicks per student. In terms of academic performance, pharmacology exam grades had a mean of 13.2 (SD = 2.2), with scores ranging from 6.6 to 18.0 and a median of 13.3. The interquartile range (IQR) was 3.1 (Table 2).

**Table 2:** Study behaviors and pharmacology exam grade of second year medical students participating to Pharmaquest

Variable	Stats / Values	Freqs (% of Valid)	Graph	Valid	Missing
Pharmacology exam grade	Mean (SD): 13.2(2.2) min ≤ med ≤ max: 6.6 ≤ 13.3 ≤ 18 IQR (CV): 3.1 (0.2)	95 distinct values		164 (100.0%)	0 (0.0%)
Class attendance	Mean(SD):12.5(10.1) min ≤ med ≤ max: 0 ≤ 8 ≤ 31 IQR (CV): 17 (0.8)	27 distinct values		164 (100.0%)	0 (0.0%)
Number of unique online resource supports downloaded	Mean (SD): 10.6 (7.7) min ≤ med ≤ max: 0 ≤ 9 ≤ 25 IQR (CV): 13 (0.7)	26 distinct values		164 (100.0%)	0 (0.0%)
Roneo lecture support subscription	1. No 2. Yes	8(4.9%) 156(95.1%)		164 (100.0%)	0 (0.0%)
Peer-tutoring mock exam attendance	Min: 0 Mean: 0.5 Max: 1	0:76(46.3%) 1:88(53.7%)		164 (100.0%)	0 (0.0%)

### Influence of learning activities on pharmacology performance

**Fig. 2:**
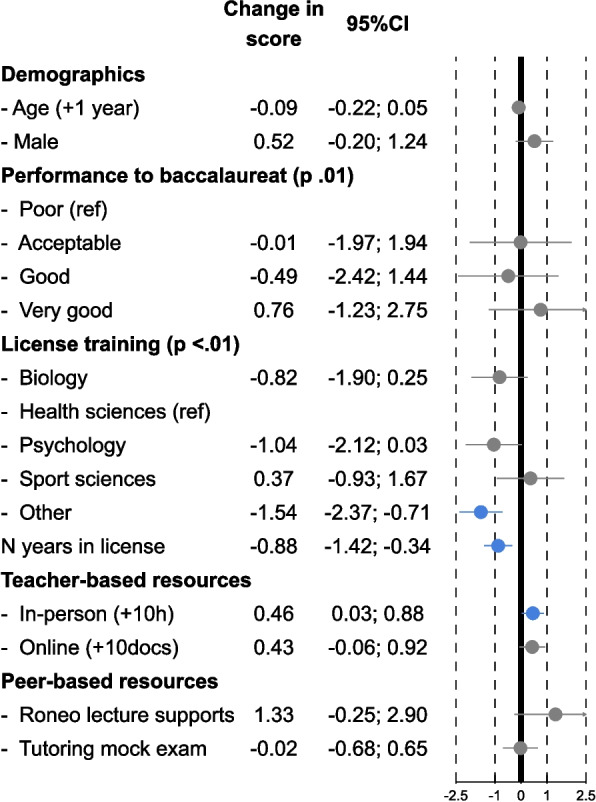
Multivariate analysis: association between learning activities, baseline characteristics, and final pharmacology exam score in second year medical students. CI: Confidence Interval

In univariate analyses (Supplementary Fig. 3), higher in-person lecture attendance was significantly associated with a higher score at the pharmacology exam, with each additional 10 h of attendance corresponding to a 0.41-point increase in the exam score (95% Confidence Interval (CI): 0.05 to 0.77). This association remained statistically significant in the multivariate model (0.46, 95%CI 0.03 to 0.88, Fig. 2). Similarly, the number of online resources downloaded showed a positive association in univariate analysis, with every 10 additional downloads associated with a 0.53-point increase in the exam score (95% CI: 0.09 to 0.97). This association lost significance in the multivariate model, with an estimated increase of 0.46 points (95% CI: –0.03 to 0.95) per 10 modules downloaded (Fig. 2). Diagnostic plots assessing model assumptions are presented in Supplementary Fig. 4. None of the demographic variables, baccalaureate performance measures, or peer-based resource usage were significantly associated with final grades (Fig. 2). Students from the “other” license category had significantly lower scores. Additionally, a longer time to complete the first license year was associated with lower pharmacology exam performance.

**Fig. 3:**
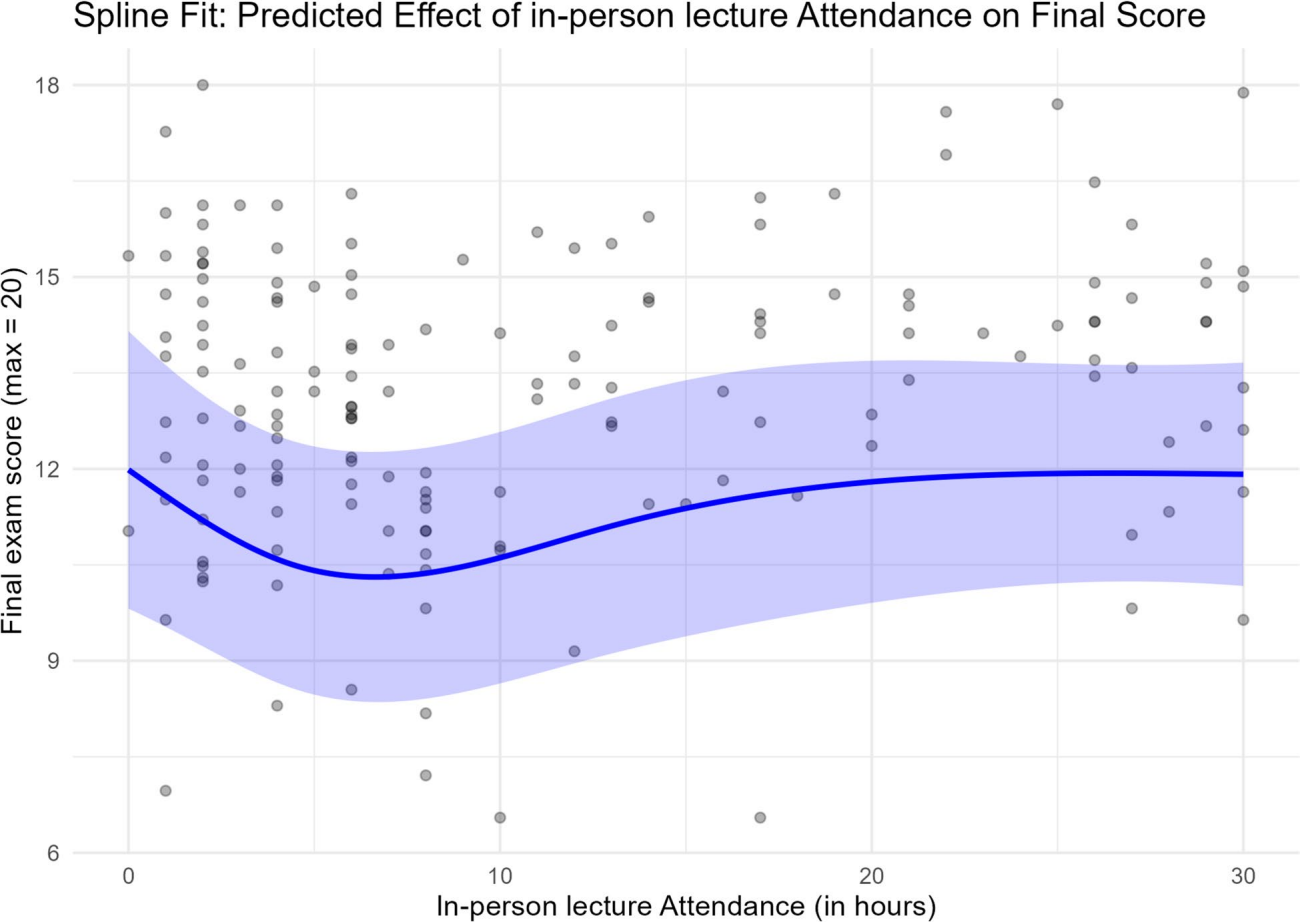
Predicted exam scores according to total in-person attendance hours using a spline-based model

To explore potential non-linear associations between in-person class attendance and exam score, we used a spline function. The spline model provided a significantly better fit than the linear model (ANOVA, *p* < 0.05), indicating a non-linear relationship. As shown in Fig. 3, predicted exam scores initially declined with increasing attendance, reaching a minimum around 10 h. Beyond this point, scores gradually increased, plateauing between 25 and 30 h of attendance. Figure 3 shows the model-predicted relationship, with its confidence interval representing uncertainty in the prediction. Supplementary Fig. 5 depicts the actual distribution of in-person lecture attendance, with a confidence interval reflecting variability in the observed data. The predicted trend is concordant with the observed attendance patterns, illustrating how the model aligns with real-world behavior.

In the random forest regression model, most influential variables were the number of years required to pass the first license year, the first license year major, and the level of distinction obtained at the baccalaureate exam. In-person lecture attendance and the number of online resources used followed in fourth and fifth positions, respectively. To better understand the contribution of each variable, Shapley values were calculated and visualized in Supplementary Fig. 6.

Supplementary Fig. 7 presents the results of univariate analyses examining the association between the in-person lecture format types and exam performance. LBL, the most prevalent approach, showed the smallest estimated gain per hour, with a significant increase in score of 0.04 points (95% CI: 0.01 to 0.08). Change in score was more important with all other tested class types, but not statistically significant, except for PBL. PBL was associated with a significant increase of 0.24 points per hour (95% CI: 0.06 to 0.42), and EBM showed a similar non-significant trend (0.29, 95% CI: 0.00 to 0.57). In a sensitivity analysis pooling all non-LBL active-learning formats together (CBL, PBL, EBM, TBL, flipped classroom, serious game) and comparing them to pure LBL, each additional hour of teaching attended was associated with a + 0.09-point change in exam score for non-LBL hours (95% CI: 0.00 to 0.18) and a + 0.07 change for LBL hours (95% CI: 0.01 to 0.13; Supplementary Fig. 7, panel B). The two estimates were not statistically distinguishable, and the comparison was constrained by the curriculum structure: of the 8 sessions involving a non-LBL active-learning element, only 2 (Major Systems revision quiz, Pharmacolympics; 5 h in total) were standalone non-LBL sessions, the remaining 6 sessions combining LBL with one or more active-learning formats. This pooled analysis should therefore be interpreted as exploratory.

Supplementary Fig. 8 illustrates a positive correlation between scores on the optional tutoring exam and the final pharmacology exam (Pearson’s *r* = 0.66, *p* < 0.0001), indicating that students who performed well on the mock exam tended to achieve higher scores on the final exam.

Total in-person lecture attendance was essentially uncorrelated with baccalaureate distinction (Spearman *ρ* = + 0.03, *p* = 0.70) and only weakly correlated with the number of years required to pass the first license year (*ρ* = + 0.12, *p* = 0.13), while both academic-background variables remained strongly correlated with the final exam score (*ρ* = + 0.33 and *ρ* = − 0.34, respectively, both *p* < 0.001), arguing against substantial confounding of the attendance estimate by prior academic capital.

## Discussion

In this prospective pilot study, pedagogical methods (in-person lecture attendance, use of online resources) tended to influence pharmacology exam performance, in second-year medical students. A non-linear relationship was observed for in-person lecture attendance, meaning that the effect of attendance on exam scores did not increase in a straight line. Prior academic background and achievements appeared as strongest factors to explain this performance. We also demonstrated the feasibility of quantitative evaluation of a pharmacology educational program, in the context of French academic medical training.

Notably, the relationship between in-person lecture attendance and exam performance was non-linear, following a U-shaped curve. Contrary to the prevailing assumption that attendance linearly enhances learning outcomes, we observed that modest increases in attendance initially corresponded to *lower* performance, with scores improving only beyond a threshold of approximately ten hours. This finding invites a more nuanced interpretation of attendance data. It may reflect compensatory behavior from struggling students—those with weaker academic preparation or less effective study strategies may attend more lectures in a passive attempt to “catch up,” without yet possessing the tools to transform that time into meaningful learning. Alternatively, students overwhelmed early in the course may attend out of obligation, but disengage cognitively [17], a phenomenon described in cognitive load theory [18]. Once students surpass a critical participation threshold—likely coinciding with improved regulation, familiarity with the material, or access to external supports—their performance begins to recover. This interpretation is consistent with models of self-regulated learning, in which students gradually adapt their strategies to optimize outcomes in response to early failure or difficulty [19, 20]. Importantly, this complex dynamic was invisible in linear models, highlighting the value of flexible, non-parametric approaches in educational research. Although the linear effect of attendance was modest (+ 0.41 points per 10 h), the magnitude predicted by the non-linear model between zero and full attendance reached ~ 1.3 points on a 20-point scale (≈ 0.6 SD of the exam score), which is large enough to move students across grade-band boundaries. In other words, the average linear coefficient under-estimates the educational significance once the U-shape is accounted for.

Building on previous work from India [9] and Sweden [10], which relied largely on self-reported behaviours, our study adds four contributions. First, we triangulated objective digital traces (LMS logs, QR-code attendance, peer-tutoring data) with individual-level exam scores, reducing recall bias. Second, we describe the determinants of pharmacology performance under the recent French curricular reform (PASS / L.AS), an under-studied context. Third, we explicitly model the non-linearity of the attendance–performance relationship using splines and machine-learning techniques rather than assuming linearity. Fourth, the analytical pipeline (publicly available with the protocol) is intended as a reproducible benchmark that institutions in any country could adapt to evaluate their own pharmacology curriculum.

Among all features analyzed, baccalaureate distinction and rapid progression through the competitive first year emerged as the most robust markers of pharmacology performance. These findings confirm longstanding concerns that pre-medical academic capital remains a major determinant of success within medical curricula, regardless of ongoing reforms. This result is supported by evidence from a recent review of medical school selection procedures in the US, which found that academic indicators such as pre-university grades consistently predict early academic performance but contribute minimally to forecasting later professional competencies—potentially perpetuating social inequities in medical training [21]. However, the absence of correlation between attendance and pre-medical academic capital indicates that these two determinants act independently on pharmacology performance, rather than one mediating the other. While the PASS/L.AS system aims to diversify access, our cohort’s composition—over 75% with high baccalaureate distinction—suggests limited success to date in mitigating structural inequities. These results echo international literature on the “Matthew effect” in education, where early academic advantages tend to accumulate and reinforce themselves over time [22]. Students who arrive with stronger academic foundations not only perform better but are often better equipped to adapt to unfamiliar pedagogies, such as active learning or self-directed study, further widening the achievement gap. To counterbalance this inertia, institutions may need to implement systematic academic bridging programs and proactive mentoring, especially for students from underrepresented or non-traditional backgrounds.

Our data also point to the importance of pedagogical format. Exploratory analyses suggest that time spent in problem-based learning (PBL) and evidence-based medicine (EBM) sessions was more strongly associated with performance improvements, rather than time spent in LBL. Although causality cannot be inferred, this aligns with meta-analytic evidence that active learning methods outperform traditional didactics in health professions education in pharmacology [23]. These formats likely promote deeper processing, greater knowledge transfer, and stronger metacognitive skills [24, 25]—all critical in mastering pharmacology. However, our findings also reflect a gap between pedagogical best practices and actual curricular delivery. Despite the demonstrated benefits of PBL and peer-led tutorials, LBL remains dominant, likely due to institutional inertia, limited faculty training in active learning, and resource constraints [26, 27]. Bridging this gap will require more than curriculum redesign; it demands structural investment in faculty development, incentives for pedagogical innovation, and ongoing evaluation of teaching effectiveness.

Interestingly, peer tutoring mock exam score was positively correlated with final pharmacology exam score (*r* = 0.66), while on the other hand, attending this mock exam was not a marker of higher performance. Peer-tutoring was restricted to this sole mock exam during the second-year of medical students in our institution, whereas it is much more developed during the first year. Also, students may be both receiving and providing peer-tutoring, respectively from older and to younger students. However, this engagement was not captured by this first iteration of Pharmaquest. It is critical to fill this analytic gap, as peer learning likely facilitates repeated exposure to exam-relevant content while fostering collaborative problem-solving, accountability, and increases motivation [28]. These benefits are well supported by social cognitive theory, which emphasizes observational learning and the value of feedback-rich environments [29]. In this context, it would be particularly relevant to investigate the role of the professor in active learning settings—whether their physical presence is essential, or if well-designed peer-led or self-directed activities could achieve comparable educational outcomes.

Finally, it is important to recognize that medical education is evolving rapidly under the influence of new technologies. Artificial intelligence tools—capable of organizing learning materials, generating questions, and supporting self-directed study—are increasingly integrated into pedagogical practice by the students themselves [30]. This ongoing technological transformation reinforces the need for systematic evaluation of teaching methods, as what is effective today may not remain so tomorrow. Continuous assessment of educational strategies will therefore be essential to ensure that pharmacology teaching adapts effectively to emerging tools and changing student needs.

Beyond the French setting, several lessons are likely to apply internationally. First, lecture attendance should not be used as a proxy of learning effort: its relationship with performance is non-linear, and large gains were obtained only above a threshold of attendance. Second, prior academic achievement remains a dominant determinant of pharmacology performance after a major curricular reform, suggesting that educational policies aimed at narrowing performance gaps must specifically support students with weaker academic capital. Third, active-learning formats (PBL, EBM, CBL) appear to produce larger per-hour gains than LBL, supporting their integration into pharmacology curricula regardless of country. Finally, the use of Learning Management System (LMS) logs and peer-tutoring data offers a low-cost, replicable framework for institutions worldwide to evaluate the impact of pedagogical choices on pharmacology learning.

There are limitations to consider. Attendance data were collected voluntarily via QR code scans, introducing possible selection and classification bias, particularly if students left the lecture before the end. Additionally, incorrect entry of student identification numbers may have resulted in data that could not be attributed to any student, leading to missing attendance records. On the other hand, it was unlikely but possible for students to check their attendance while being absent, should they have recorded the external link provided by the QR code. This behavior would have been problematic if students felt they could benefit from fictively attend lectures. Comprehensive explanations through direct communication and information supports made it clear that students should not expect any benefit from either participating to Pharmaquest nor masquerading pedagogical methods. Two alternative explanations should be considered for the attendance–performance association. First, reverse causation: students who already feel well-prepared may be more inclined to attend lectures, and conversely struggling students may disengage, producing an attendance signal that partly reflects rather than causes performance. Second, self-selection: highly motivated students may simultaneously attend more lectures and engage in additional study activities not measured here (private revision, AI-assisted study), so attendance may act as a marker of broader engagement. The weak correlation between attendance and pre-medical academic capital observed in our data (*ρ* = + 0.03 with baccalaureate distinction and *ρ* = + 0.12 with the number of years to pass the first license year; see Results) makes simple confounding by prior achievement unlikely, but residual unmeasured confounding by motivation cannot be excluded. Both mechanisms warrant explicit testing in future cluster-randomised or instrumental-variable designs. The study was conducted at a single institution, with relatively limited variation in academic background, which restricts generalizability. Key psychosocial variables—such as socioeconomic status, mental health, and study habits—were not captured, though they likely moderate the effects of both pedagogy and participation, measured through objective metrics in the study [31]. Our analyses remain observational and cannot determine causality. The associations observed may reflect underlying motivation or study habits rather than the direct effect of class attendance itself. External replication will be needed to improve the generalizability of our findings. Our sample size (*n* = 164) limited the complexity and statistical power of the models. Future studies should employ experimental or quasi-experimental designs to evaluate the direct impact of pedagogical interventions. Looking ahead, multi-institutional studies could test whether these findings hold across diverse curricula and student populations, particularly under the newer PASS/L.AS framework. Finally, qualitative studies exploring students lived experiences of pharmacology learning—especially in relation to motivation, participation, and the perceived value of various teaching formats—could provide crucial context to our quantitative findings. Such studies could also help identify potential barriers to attendance or students’ preferred study strategies. For instance, some students may perceive self-paced learning as more efficient than lecture-based learning (LBL), especially when sessions are not directly aligned with exam content or clinical application. Importantly, the goal of pharmacology education is not to promote attendance for its own sake, but to support the development of competent future professionals. Attendance should be encouraged only insofar as it meaningfully enhances student learning.

Furthermore, integrating an international dimension in future studies would add critical value. Medical education systems vary considerably in terms of curricular structure, cultural attitudes toward learning, and access to academic support [32]. Cross-national comparisons could help identify universal drivers of student success versus those shaped by local educational policies or sociocultural norms. For example, investigating how active learning is perceived and implemented across different countries could reveal whether certain pedagogical innovations are more effective—or more equitably impactful—in specific contexts. Incorporating international cohorts would also enhance the external validity of findings and foster the development of globally informed, culturally sensitive educational strategies, especially crucial in an increasingly interconnected medical community.

## Conclusions

This pilot study demonstrated the feasibility and value of quantitatively assessing a pharmacology education program among French medical students, revealing that exam success was most associated with prior academic background and achievements, and that pedagogical methods had possible importance, provided the potential non-linear relationship is handled appropriately. These results suggest that more personalized and inclusive teaching strategies, taking into account the diversity of backgrounds, learning behaviours and educational contexts of students, could be worth promoting. Personalization should recognize that some students benefit more from independent study with trustworthy resources, while others may require more guided, interactive learning. A key limitation, however, is the absence of data on students’ individual study habits, which likely play a major role in academic performance. Broader replication with inclusion of study habits, long-term follow-up, and international collaboration are essential next steps to identify robust determinants of success and to inform equitable, evidence-based educational programs.

## Supplementary Information

Supplementary Material 1: Supplementary Methods [33].

Supplementary Material 2: Supplementary Table 1. In-person lecture characteristics.

Supplementary Material 3: Supplementary Fig. 1. Evolution of Class attendance over the semester. Nov: November, Oct: October, Sept: September. Supplementary Fig. 2. Student interaction with online resources. Panel A: Number of available resources. Panel B: Number of clicks. Supplementary Fig. 3. Univariate analyses: Association between learning activities, baseline characteristics, and final pharmacology exam score in second year medical students. CI: Confidence Interval. Supplementary Fig. 4. Assumption checker for multivariate analysis. Supplementary Fig. 5. Exam scores according to total in-person attendance hours using a spline-based model. Supplementary Fig. 6. SHAP Values Analysis: Direction and Magnitude of Associations with Exam Scores. Supplementary Fig. 7. Results of univariate analyses examining the association between the in-person lecture format types and exam performance. Supplementary Fig. 8. Correlation between scores on the optional tutoring exam and the exam.

## Data Availability

All datasets and code generated or analyzed during the current study are freely available from the corresponding author upon reasonable request.

## Abbreviations

AIC: Akaike Information Criterion
BMCT: Bases Moléculaires, Cellulaires et Tissulaires des Thérapeutiques (Molecular, Cellular, and Tissue Bases of Drug Treatments)
CBL: Case-based learning
CEMU: Centre d’Enseignement Multimédia Universitaire (Appears in author affiliations)
CI: Confidence Interval
CIRNEF: Centre interdisciplinaire de Recherche Normand en Education et Formation (Appears in author affiliations)
CV: Coefficient of Variation
df: Degrees of freedom
EBM: Evidence-based medicine
GABA: Gamma-aminobutyric acid (Appears in lesson title: “GABA, glutamate”)
GIP: Groupement d’Intérêt Public (Appears in author affiliations)
INSERM: Institut national de la santé et de la recherche médicale (Appears in author affiliations)
IQR: Interquartile range
L.AS: Licence avec Accès Santé
L2: Second-year students (or second license year)
L3: Third-year students
LBL: Lecture-based learning
MCQs: Multiple-choice questions
MD: Medical Doctor
MMOPK: Medicine, Midwifery, Odontology, Pharmacy, and Physiotherapy
MPH: Master of Public Health
MSc: Master of Science
PASS: Parcours Accès Santé Spécifique
PBL: Problem-based learning
PD: Pharmacodynamics (Appears in lesson title: “PK PD Principles”)
PhD: Doctor of Philosophy
PharmD: Doctor of Pharmacy
PK: Pharmacokinetics (Appears in lesson title: “PK PD Principles”)
RAAS: Renin-Angiotensin-Aldosterone System (Appears in lesson title)
SBAs: Single Best Answers
SD: Standard deviation
SHAP: Shapley Additive Explanations
TBL: Team-based learning
UNICAEN: Université Caen Normandie
US: United States
VIF: Variance Inflation Factors
VSAQs: Very Short Answer Questions
