# Supplementary material for: Influence of learning activities and background characteristics on pharmacology exam success in second-year medical students at a French university: the Pharmaquest study
Source: BMC Med Educ. 2026 May 18;26:1102. doi: 10.1186/s12909-026-09454-7 (PMC13348608; doi:10.1186/s12909-026-09454-7)
Supplement: Supplementary file 1 — Supplementary Material 1: Supplementary Methods [33]. [file 12909_2026_9454_MOESM1_ESM.docx]

**Supplementary Data**

**Supplementary Methods**

Splines: The optimal number of df was determined by comparing models with df ranging from 3 to 7 using model selection criteria, specifically the Akaike Information Criterion (AIC). The model with 3 degrees of freedom yielded the lowest AIC and was selected as the final model for analysis.

The main spline model incorporated the duration of class attendance, the number of modules attended, and other covariates used in the main outcome as predictor variables. The spline term for class attendance was modeled to allow for a flexible, non-linear relationship. Additionally, the spline model was compared with a simple linear regression model to evaluate whether the spline approach provided a better fit. This comparison was done using an analysis of variance (ANOVA).

Random Forest and SHAP values: These values provide an interpretable measure of how much each feature contributed to individual predictions, thereby enhancing transparency in model interpretation[33]. The results were visualized using summary plots, where each point represents a student, and the position along the x-axis reflects the impact (positive or negative) of a specific variable on the predicted grade. The color gradient illustrates the actual value of the variable for each student, enabling a nuanced understanding of how different feature levels influence predictions across the dataset.
