# Supplementary material for: Influence of learning activities and background characteristics on pharmacology exam success in second-year medical students at a French university: the Pharmaquest study
Source: BMC Med Educ. 2026 May 18;26:1102. doi: 10.1186/s12909-026-09454-7 (PMC13348608; doi:10.1186/s12909-026-09454-7)
Supplement: Supplementary file 2 — Supplementary Material 2: Supplementary Table 1. In-person lecture characteristics. [file 12909_2026_9454_MOESM2_ESM.docx]

**Supplementary Tables**

**Supplementary Table 1: In-person lecture characteristics**

| Class characteristics | |  | Type of class | | | | | | |
| --- | --- | --- | --- | --- | --- | --- | --- | --- | --- |
| Lesson name | Duration (hours) |  | Case-Based Learning | Evidence-Based Medicine | Flipped Classrooms | Lecture Based Learning | Problem-Based Learning | Team-Based Learning | Serious Game |
| Introduction | 1 |  | 0 | 0 | 0 | 1 | 0 | 0 | 0 |
| Proper use of drugs | 1 |  | 0 | 0 | 0 | 1 | 0 | 0 | 0 |
| GABA, glutamate | 2 |  | 0 | 0 | 0 | 1 | 0 | 0 | 0 |
| Autonomous nervous system | 2 |  | 1 | 0 | 0 | 1 | 1 | 0 | 0 |
| RAAS | 1 |  | 1 | 0 | 0 | 1 | 0 | 0 | 0 |
| Clinical development | 1 |  | 1 | 1 | 0 | 1 | 0 | 0 | 0 |
| Dopamine | 1 |  | 0 | 0 | 0 | 1 | 0 | 0 | 0 |
| Serotonin, histamine | 1 |  | 0 | 0 | 0 | 1 | 0 | 0 | 0 |
| Major Systems Quiz | 2 |  | 0 | 0 | 1 | 0 | 0 | 1 | 1 |
| PK PD Principles (1/2) | 2 |  | 0 | 0 | 0 | 1 | 0 | 0 | 0 |
| PK PD Principles (2/2) | 1 |  | 0 | 0 | 0 | 1 | 0 | 0 | 0 |
| Treatment Individualization | 1 |  | 0 | 0 | 0 | 1 | 0 | 0 | 0 |
| Special populations (1/2) | 2 |  | 0 | 0 | 0 | 1 | 0 | 0 | 0 |
| Special populations (2/2) | 1 |  | 0 | 0 | 0 | 1 | 0 | 0 | 0 |
| Administration Routes | 1 |  | 0 | 0 | 0 | 1 | 1 | 0 | 1 |
| Drug-Induced Organ Damage (1/2) | 1 |  | 0 | 0 | 0 | 1 | 0 | 0 | 0 |
| Drug-Induced Organ Damage (2/2) | 1 |  | 0 | 0 | 0 | 1 | 0 | 0 | 0 |
| Cancer drugs | 1 |  | 0 | 0 | 0 | 1 | 0 | 0 | 0 |
| Pharmacovigilance | 2 |  | 1 | 0 | 0 | 1 | 0 | 0 | 0 |
| Therapeutic education | 1 |  | 0 | 0 | 0 | 1 | 0 | 0 | 0 |
| Addictovigilance | 1 |  | 0 | 0 | 0 | 1 | 0 | 0 | 0 |
| Critical Analysis, Level of Evidence | 2 |  | 0 | 1 | 0 | 1 | 1 | 0 | 0 |
| Analgesics | 2 |  | 0 | 0 | 0 | 1 | 0 | 0 | 0 |
| Pharmacolympics | 3 |  | 0 | 0 | 0 | 0 | 0 | 1 | 1 |

Case-based Learning engages students in discussion of specific scenarios that resemble or typically are real-world examples,

The flipped Classroom is an educational approach that reverses the nature of learning activities in class and at home. Traditional learning roles are modified according to the expression "lessons at home and homework in class."

Lecture Based Learning refers to traditional passive lecture.

Problem-based learning is a student-centered approach in which students learn about a subject by working in groups to solve an open-ended problem. This problem is what drives the motivation and the learning.

Team-based learning is a pedagogical strategy that engages student knowledge through individual testing and group collaboration. Following individual answers, students join teams and work through problems, appealing when they are incorrect.

A serious game is an activity that combines a "serious" intention—such as educational—with playful elements.
