# Supplementary material for: Influence of learning activities and background characteristics on pharmacology exam success in second-year medical students at a French university: the Pharmaquest study
Source: BMC Med Educ. 2026 May 18;26:1102. doi: 10.1186/s12909-026-09454-7 (PMC13348608; doi:10.1186/s12909-026-09454-7)
Supplement: Supplementary file 3 — Supplementary Material 3: Supplementary Fig. 1. Evolution of Class attendance over the semester. Nov: November, Oct: October, Sept: September. Supplementary Fig. 2. Student interaction with online resources. Panel A: Number of available resources. Panel B: Number of clicks. Supplementary Fig. 3. Univariate analyses: Association between learning activities, baseline characteristics, and final pharmacology exam score in second year medical students. CI: Confidence Interval. Supplementary Fig. 4. Assumption checker for multivariate analysis. Supplementary Fig. 5. Exam scores according to total in-person attendance hours using a spline-based model. Supplementary Fig. 6. SHAP Values Analysis: Direction and Magnitude of Associations with Exam Scores. Supplementary Fig. 7. Results of univariate analyses examining the association between the in-person lecture format types and exam performance. Supplementary Fig. 8. Correlation between scores on the optional tutoring exam and the exam. [file 12909_2026_9454_MOESM3_ESM.zip › SHAP_legend.docx]

**Legend for feature values in the SHAP plot:**

- **Years to pass 1^st^ year**: 0 (via transition program) to 3 years
- **License category**:
  0 = Health sciences, 1 = Biology, 2 = Other, 3 = Psychology, 4 = Sports science
- **Baccalaureate mention**:
  0 = No mention, 1 = Fairly good, 2 = Good, 3 = Very good
- **Class hours attended**: Continuous, from 0 to 31 hours
- **Lesson files downloaded**: Continuous, from 0 to 25 files
- **Age (years)**: Continuous, from 18 to 36 years
- **Gender**: 0 = Female, 1 = Male
- **Participation in tutoring**: 0 = No, 1 = Yes
- **Participation in Roneo**: 0 = No, 1 = Yes

*In the plot, "Low" and "High" refer to the relative values of each feature within the dataset — with "Low" indicating lower values or absence (e.g., no participation), and "High" indicating higher values or presence.*
