# Supplementary material for: Influence of learning activities and background characteristics on pharmacology exam success in second-year medical students at a French university: the Pharmaquest study
Source: BMC Med Educ. 2026 May 18;26:1102. doi: 10.1186/s12909-026-09454-7 (PMC13348608; doi:10.1186/s12909-026-09454-7)

	Change in score	95%CI
**Class type (+1h)**		
- Case-based learning	0.15	0.00; 0.30
- Evidence Based Med.	0.29	0.00; 0.57
- Flipped Classroom	0.07	-0.28; 0.41
- Lecture based learning	0.04	0.01; 0.08
- Problem-based learning	0.24	0.06; 0.42
- Serious game	0.14	-0.13; 0.41
- Team-based learning	0.07	-0.28; 0.41

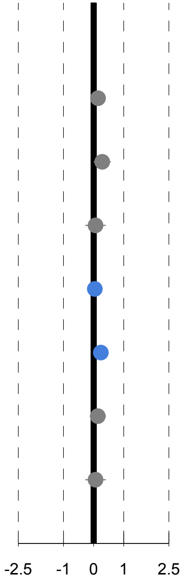

Supplement: Supplementary file 3 — Supplementary Material 3: Supplementary Fig. 1. Evolution of Class attendance over the semester. Nov: November, Oct: October, Sept: September. Supplementary Fig. 2. Student interaction with online resources. Panel A: Number of available resources. Panel B: Number of clicks. Supplementary Fig. 3. Univariate analyses: Association between learning activities, baseline characteristics, and final pharmacology exam score in second year medical students. CI: Confidence Interval. Supplementary Fig. 4. Assumption checker for multivariate analysis. Supplementary Fig. 5. Exam scores according to total in-person attendance hours using a spline-based model. Supplementary Fig. 6. SHAP Values Analysis: Direction and Magnitude of Associations with Exam Scores. Supplementary Fig. 7. Results of univariate analyses examining the association between the in-person lecture format types and exam performance. Supplementary Fig. 8. Correlation between scores on the optional tutoring exam and the exam. [file 12909_2026_9454_MOESM3_ESM.zip › Supplementary_figure_7.pdf]
